# Practice and lived experience of menstrual exiles (*Chhaupadi*) among adolescent girls in far-western Nepal

**DOI:** 10.1371/journal.pone.0208260

**Published:** 2018-12-10

**Authors:** Prabisha Amatya, Saruna Ghimire, Karen E. Callahan, Binaya Kumar Baral, Krishna C. Poudel

**Affiliations:** 1 Department of Public Health, Central Institute of Science and Technology (CIST) College, Pokhara University, Kathmandu, Nepal; 2 Agrata Health and Education (AHEAD)-Nepal, Kathmandu, Nepal; 3 School of Community Health Sciences, University of Nevada Las Vegas, Las Vegas, NV, United States of America; 4 Department of Biochemistry, Nepal Medical College and Teaching Hospital, Kathmandu, Nepal; 5 Department of Health Promotion and Policy, School of Public Health and Health Sciences, University of Massachusetts Amherst, Amherst, MA, United States of America; London School of Hygiene and Tropical Medicine, UNITED KINGDOM

## Abstract

**Background:**

Menstrual exile, also known as *Chhaupadi*, is a tradition of “untouchability” in far-western Nepal. Forbidden from touching other people and objects, women and girls are required to live away from the community, typically in a livestock shed, during menstruation. We assessed the lived experiences of *Chhaupadi* among Nepalese adolescent girls in the far-western Achham district of Nepal, observed the safety and sanitation of their living spaces during *Chhaupadi*, and assessed the perceptions of local adult stakeholders towards the practice of *Chhaupadi*.

**Methods:**

We collected data from 107 adolescent girls using a self-administered survey in two local schools in Achham. We also conducted a focus group discussion with seven girls, held key informant interviews, and observed the girls’ living spaces during *Chhaupadi*, using a checklist. Descriptive statistics of the quantitative survey and thematic analyses of qualitative interviews are presented.

**Results:**

The majority of the girls (n = 77, 72%) practiced exile, or *Chhaupadi*, during their menstruation, including 3 (4%) exiled to traditional *Chhau* sheds, 63 (82%) to livestock sheds, and 11 (14%) to courtyards outside their home. The remaining girls (n = 30, 28%) stayed inside the house, yet practiced some form of menstrual taboos. Of the 77 observed living spaces where the girls stayed during exile, only 30% (n = 23) had a toilet facility. Most exiled girls (97.4%) were restricted from eating dairy products. Participants reported having various psychological problems, including lonliness and difficulty sleeping while practicing *Chhaupadi*. Three of the girls were physically abused; nine were bitten by a snake. Notably high proportions of the living spaces lacked ventilation/windows (n = 20, 26%), electricity (n = 29, 38%), toilets (n = 54, 70%) and a warm blanket and mattress for sleeping (n = 29, 38%). Our qualitative findings supported our quantitative results.

**Conclusions:**

*Chhaupadi* has been condemned by human rights organizations. While the government has banned the practice, implementation on the ban is proceeding slowly, especially in far-western Nepal. Thus, as a temporary measure, public health professionals must work towards promoting the health and safety of Nepalese women and girls still practicing *Chhaupadi*.

## Introduction

Menstruation, a natural biological phenomenon common to most females that marks the beginning of womanhood, is inextricably linked to the sustainability of mankind. Ironically, in many parts of the world, menstruation is steeped in silence, myths, taboos and even stigma [1,2]. In South Asian countries, a range of restrictions, regarding food, school attendance, household chores and social events, are placed on menstruating women and girls [2,3]. Additionally, research suggests that menstrual hygiene practices are poor and many girls miss school due to menses throughout Africa and Asia [3].

In the Himalayan country of Nepal, menstruation is considered taboo, an event of stigma and sin [4,5]. Menstruating women and girls are considered impure and untouchable; as such, they are isolated from daily household activities and social events [4,6]. Anything touched by a menstruating woman is deemed impure and needs to either be discarded or purified in some way [4,6]. Consequently, menstruating women and girls are forbidden from physically touching certain people (specifically males who have undergone the sacred rituals of “*Bratabandha*”), as well as livestock, plants, kitchen items and drinking water sources, which limits access to food, drinking water, and other necessities [4]. The perception of menses as impure is so steeped in Nepali culture and tradition that a yearly festival called “Rishi Panchami” is observed for one day in August by all menstruating women and girls, who purify themselves with water, prayer, and fasting for the “sins” they committed while menstruating [7].

Although prevalent throughout Nepal, the social taboo against menses is harshest particularly in far-western Nepal, where menstruating women and girls are banished to a makeshift hut or livestock shed [4,6]. Menstrual exile in this region is called “*Chhaupadi*”, derived from two words: “*Chhau*” meaning menstruation and “*padi*” meaning women [4]. The temporary shelter where menstruating women and girls traditionally reside, called the *Chhau* shed [6], has been criticized for being unhygienic, exposed, unsafe, and lacking basic necessities [6,8]. A short video by the Guardian (http://bit.ly/2hPS6MI) illustrates the challenges facing women and girls who practice *Chhaupadi* [7]. Customarily, banishment to the *Chhau* shed, or in more recent times, a livestock shed, occurs with each menstruation cycle, generally lasting four consecutive days. Moreover, girls experiencing menses for the first time are expected to remain in the *Chhau* or livestock shed for at least 14 days [6]. Compounding the situation is that if the young women and girls encounter any health issues while in menstrual exile, they are expected to wait until their menstruation is completed before seeking medical care [9].

Notably, the practice of *Chhaupadi* is not limited to times of menstruation but also to the time of childbirth whereby delivery must take place in the unhygienic shed [10]. Women and their fragile newborns are compelled to stay in exile for 10–14 days post-delivery [10]. Consequently, *Chhaupadi* during childbirth can lead to both maternal and infant death arising from excessive bleeding, septic shock, and even relatively normal complications that are not resolved due to lack of access to health care [11]. Although exact figures of maternal and child health consequences due to *Chhaupadi* are unknown, neonatal and maternal mortality is high in the far-western regions where *Chhaupadi* is common [8,12]. Moreover, reproductive tract infections and utero-vaginal prolapse are the leading causes of maternal morbidity in regions practicing *Chhaupadi* [13,14].

The practice of *Chhaupadi* requires urgent public health attention. Temporarily living in an unhygienic livestock shed or traditional *Chhau* shed increases the likelihood of diarrhea and dehydration, hypothermia, reproductive tract and urinary tract infections [6,14]. Moreover, the mental health of women and girls is impacted by feelings of abandonment, insecurity, guilt, and humiliation for being “impure” and “untouchable” [6]. Worse still, deaths have been reported from poisonous snake and scorpion bites as well as wild animal attacks on women and girls residing in menstrual exile sheds [6].

In the wake of three highly publicized deaths within ten months among women and girls practicing *Chhaupadi* [15], the Nepali Parliament enacted a new law in August 2017 criminalizing the *Chhaupadi* practice and imposing a fine and/or a three-month jail sentence for anyone forcing a woman to follow the custom [15,16]. The new law, set for enforcement beginning in August 2018 [16], is definitely welcome news. However, advocates of the ban believe that enacting the law alone will not solve the problem; the real challenge will be enforcing it [16]. A prior well-intended social change campaign, led primarily by educated members of the government and non-governmental organizations, engaged in the practice of physically destroying traditional *Chhau* sheds, (those built exclusively for menstrual exile). They then triumphantly declared certain villages as “*Chhaupadi*-free” [11]. However, knocking down the shed alone didn’t stop the practice or change sociocultural beliefs. Sheds were either rebuilt or menstruating women and girls were exiled to even more unhygienic and dangerous structures, sheds shared with livestock [6,8,17,18]. Moreover, *Chhaupadi* had already been banned by the Nepalese Supreme Court in 2005 [6], deemed a discriminatory practice that challenges fundamental human and women’s rights. Yet, the persistence of the practice of *Chhaupadi* in the far-western region of Nepal, suggests that more than campaigns and laws will be required for elimination [6] because the taboo against menses is so firmly entrenched. One report from a United Nation’s field bulletin, based on a personal communication with the local Women’s Development Office, estimated that in the Achham district of far-western Nepal, over 95% of women and girls practice menstrual exile, or *Chhaupadi* [6].

Despite the threat this practice poses to the health and well-being of women and girls, limited research has been conducted to assess the practice of *Chhaupadi* in Nepal. Searching PubMed using the word “*Chhaupadi*” resulted only in a study assessing the factors of reproductive health problems related to *Chhaupadi* [14] and an essay from an international volunteer’s stay in the far-western region [4]. Specifically, very little is known about the experience of adolescent girls, defined by the World Health Organization as individuals aged 10–19 years [19], practicing *Chhaupadi*. Their viewpoints are important to evaluate; they are recently introduced to nature’s phenomenon of menstruation and newly experiencing the practice of *Chhaupadi*. These young girls represent the future of Nepal and may hold the key to eliminating *Chhaupadi*. Thus, it is essential to evaluate their perceptions and lived experience of *Chhaupadi*.

Therefore, the overarching objective of our study was to assess the practice of *Chhaupadi* with four specific aims: i) to assess the prevalence of *Chhaupadi* practice among adolescent girls; ii) to observe the physical conditions and sanitation of the living spaces during *Chhaupadi*; iii) to assess the lived experience of *Chhaupadi* among adolescent girls; and iv) to assess the perceptions of local stakeholders towards *Chhaupadi*.

## Methods

### Study design and setting

A cross-sectional mixed-methods study design was used. Because the prevalence of *Chhaupadi* practice is high in Achham [6], one of Nepal’s 75 districts, we randomly selected one of Achham’s 75 villages, (locally called Village Development Commitees, or VDCs), to conduct this study. Located in the far-western region of the country, Achham is a remote and underdeveloped district. The literacy rate (ages 5+) for Achham district is 56% (71% and 43% among males and females, respectively), compared to the national literacy rate in Nepal of 66% (75% and 57% among males and females, respectively) [20]. Being nestled in the mountains, the Achham district has access to tapped/piped water in households somewhat more than national average, 56% in Achham compared to 48% across the nation. [20]. However, household electricity and household toilet prevalence are much lower in Achham: Only 18% of households have electricity and 48% have toilets compared to Nepal’s national prevalance of 67% electricity and 62% toilet in households [20]. It has been suggested that the tradition of *Chhaupadi* may have originated in Achham since the word *Chhaupadi* is derived from the local Rawte language [21]. According to the Nepali Census of 2011, our selected village had 621 households, a total population of 3124, and 421 girls between the ages of 10–19 [20].

### Ethics and consent

Ethical approval for the study, including obtaining consent from the students (without parental consent) and school principals, was provided by the Ethical Review Board at the Nepal Health Research Council. Permission was granted from the Principals of the participating schools prior to the study. Participants were provided a detailed explanation of the purpose and scope of the study, as well as an informed consent form. Written consent was taken from each participant before data collection, before focus group discussion, and before interviews. Participation was voluntary and the identity of the participants as well as the village was kept confidential.

### Study procedure

A sample size of 104 was estimated for our survey with Decision Analyst software by using the *Chhaupadi* prevalence of 95% in Achham district [6], 95% confidence intervals, 5% precision level, and a total population of girls, 10–19 years, in the target village as 421 [20].

There are total of six schools in the selected VDC with an overall 73.6% school attendance rate for females aged 5–25 years; however, only two schools had grades eight and above, and were thus selected for inclusion in our study [22]. In these two schools, a total of 132 girls were enrolled in grades 8–12. Adolescent girls at one of the two schools, ages 12–19 years old, who resided in the selected village, self-reported having menarche, and were present on the day of data collection were eligible for inclusion. We recruited all of the 107 eligible girls for the quantitative survey. After explaining the scope and objectives of the study, we had no refusals to participate. Of the 107 participants, 30 girls reported staying inside their home during menstruation, while still practicing menstrual taboos. For the remaining 77 girls, the first author, guided by student volunteers, conducted direct observation of their living spaces (*Chhau* sheds, livestock sheds, courtyards) during menstruation with a 13-point checklist.

A single focus group discussion was held with a small group of seven consenting student participants. Three key informant interviews were conducted individually with one school teacher, one female community health volunteer, and a representative from a local organization with over 13 years of related work experience. The students and the school teacher were selected by purposive sampling on first-come basis, depending upon their interest and willingness to participate. The female community health volunteer and the representative from the local organization were selected by referral made by the school teacher. Both the focus group and the key informant interviews were conducted at the local school in the Nepali language by the first author, a native Nepali speaker.

### Data collection

#### Survey tool

Self-administered surveys were completed by all participants. The survey tool was developed by the research team, using the Nepali language, based on an extensive literature review [23, 24]. For quality assurance, the survey tool was piloted among ten adolescent girls from a village in a nearby district with high prevalence of *Chhaupadi*. No major changes in the content was made following the pretest.

Demographic information on age, ethnicity, educational status, marital status, participant’s occupation (if any), family’s occupation, and monthly household income were collected. The categories for ethnicity were adopted from the ‘caste/ethnic groupings’ in Nepal’s Health Management Information System [25]. Demographic information was compared between those who practiced menstrual exile and those who remained inside the home.

For quantifying characteristics of *Chhaupadi* practice among the 77 girls who practiced menstrual exile, survey questions assessed their usual living, eating, and toileting arrangements during *Chhaupadi*, any dietary restrictions, and hygiene conditions. Participants were asked if they generally attended school and read books while menstruating. Self-reported physical and mental health problems, typically experienced while practicing *Chhaupadi* were also assessed in the survey.

#### Observation checklist

A 13-item observation checklist was developed by the study team to assess the physical condition and sanitation of the *Chhau* or livestock sheds, or exterior courtyards where participants lived during their menstrual exile. Items included water sources, ventilation, secure doors/windows, and electricity in the areas. Other items included the provision of a toilet and its distance from the shed, distance to the nearest house, distance to the nearest tap or water source, type of sleeping arrangements, availability of a place to dry clothes, and brightness of the shed.

#### Qualitative data

The focus group discussion aimed to explore the lived experiences of *Chhaupadi* and the meanings given to those experiences, following a phenomenological framework [26]. The phenomenological approach explores the essence of lived experiences of the participants about a phenomenon in the contexts or situations in which they experienced it [27]. Using the phenomenological approach, a researcher sets aside his/her own opinion and rather seeks to establish the meaning of a phenomenon from the views of the participants [26]. The discussion lasted for one hour and questions revolved around reasons for following *Chhaupadi*, “do’s and don’ts” during menstruation, nutritional restrictions during *Chhaupadi*, and willingness to follow the *Chhaupadi* practice. The key informant interviews, aimed to explore the past and present situation of *Chhaupadi* in the area, revolved around perceptions towards *Chhaupadi*, the problems of *Chhaupadi*, challenges and ideas on the way forward.

### Data processing and analysis

The quantitative data were managed in Microsoft Excel. To ensure the accuracy and quality of data entry, five percent of the total data were randomly selected and manually rechecked for correctness. This process was repeated until no errors were found. Statistical analyses were performed in IBM SPSS22 for Windows (SPSS Inc. Chicago IL, USA). Descriptive statistics calculated included frequencies, proportions, means, and standard deviations (SD).

The focus group discussion and the key informant interviews were transcribed verbatim and coded using thematic analysis [28]. Analysis was independently conducted by two members of the research team, one of whom was the interviewer who collected the data. After reading and reviewing the transcripts, researchers identified patterns in meaning, concepts, and themes.

## Results

### Quantitative findings

#### Demographic characteristics of study participants

There were no statistically significant differences in demographic characteristics of participants who practice menstrual exile and those who did not. The mean age of participants was 15 years old and ranged from 13–19. All participants were Hindu; most were from the Upper Caste and unmarried. Most participants were in high school and came from a family involved in agriculture; mean household monthly income was $143 (Table 1).

**Table 1:** Demographic characteristics of 107 study participants by menstrual exile practice.

	Practice Menstrual Exile?				
	YES (n = 77)		NO (n = 30)		p-value
	Mean	± SD	Mean	± SD	
**Age in years**	15.9	±1.3	15.5	±1.7	0.348*
**Family’s monthly income, $**	141.1	±99.8	148.3	±103.4	0.748*
** **	**Frequency**	**%**	**Frequency**	**%**	
**Ethnicity**	** **	** **		** **	0.780
Dalit	13	16.9	6	20	
Upper Caste	64	83.1	24	80	
**Educational status**	** **	** **	** **	** **	0.065
Middle school	25	32.5	11	36.7	
High school	52	67.5	19	63.3	
**Marital status**					0.575
Married	4	5.2	0	0	
Unmarried	73	94.8	30	100	
**Family’s primary occupation**					0.143
Agriculture	62	80.5	20	66.7	
Non-agriculture	15	19.5	10	33.3	

*p-value from t-test; all other p-values from Fisher's exact test

#### Practice of *Chhaupadi*

The majority of the girls (n = 77, 72%) practiced exile, or *Chhaupadi*, during their menstruation. The remaining girls (n = 30, 28.0%) stayed inside the house yet practiced menstrual taboos. Among the girls practicing menstrual exile (n = 77), over half of the participants lived in a livestock shed, ate outside their home, and defecated in open spaces (Table 2). Participants were restricted from eating dairy products. Hygiene was poor among the participants as none of the girls bathed daily; the majority bathed only once during the menstruation cycle. The majority of the girls used old clothes to absorb the flow of menstrual blood (Table 2).

**Table 2:** Characteristics of the practice of *Chhaupadi* among study participants (N = 77).

	Frequency	%
**Living space during menstruation**		
*Chhau* shed	3	3.9
Livestock shed	63	81.8
Courtyard	11	14.3
**Eating space during menstruation**		
Place stayed during menstruation	29	37.7
Outside home	48	62.3
**Place of excretion during menstruation**		
Temporary toilet	25	32.5
Open place	52	67.5
**Food prohibited during menstruation**		
Milk and milk products	75	97.4
Fruits and vegetables	2	2.6
**Frequency of bath during menstrual cycle**		
Once	54	70.1
2–3 times	12	15.6
More than 3 times	11	14.3
**Material used to absorb menstrual blood**		
Homemade pad	8	10.4
Clothes	69	89.6
**Frequency of changing pad or cloths**		
Every 6 hours	41	53.3
Less than 6 hours	9	11.7
More than 6 hours	27	35.1
**Place for drying washed clothes**		
Outside home in sunlight	75	97.4
Inside the living spaces	2	2.6

#### Problems experienced during *Chhaupadi*

The majority of the participants were allowed to read books and attend school (Table 3). Participants reported having various physical and psychological concerns while practicing *Chhaupadi*. Three (3.9%) of the adolescent girls were physically (but not sexually) abused, and nine (11.7%) were bitten by a snake (Table 3).

**Table 3:** Problems experienced by participants during *Chhaupadi* (N = 77).

Problems	Frequency	%
**Attended school during menstruation (yes)**	70	90.9
**Read books during menstruation (yes)**	71	92.2
**Physical issues encountered**		
Physical abuse	3	3.9
None	74	96.1
**General health problems** *		
Lower abdominal and back pain	20	26.0
Headache	21	27.3
Diarrhea	15	19.5
Dehydration	12	15.6
Problem with urination	9	11.7
Fever	5	6.5
**Psychological problems** *		
Loneliness	16	20.8
Lack of interest	14	18.2
Irritation	8	10.4
Sleep disturbance	9	11.7
Skin problems*		
Dry skin	5	6.5
Itch and rashes	13	16.9
**Reproductive problems**		
Vaginal inflammation	11	14.3
None	66	85.7
Environmental problems*		
Snake bite	9	11.7
Insect bite	12	15.6
**Problems due to cold (yes)**	64	83.1

*participants selected all that applied.

### Observation of living spaces during *Chhaupadi*

Observation of the living spaces during menstruation was made for the 77 (72%) girls who practiced menstrual exile. A high proportion of the observed structures lacked ventilation/windows (n = 20, 26%), electricity (n = 29, 38%), and toilets (n = 54, 70%) (Table 4). Similarly, 38% (n = 29) of the living spaces did not have a warm blanket and a mattress for the girls to sleep on.

**Table 4:** Findings from observation of menstrual exile living spaces (N = 77).

	n	%
No availability of ventilation/windows	20	26.0
No availability of lock in doors and windows	9	11.7
No availability of electricity	29	37.7
No brightness	27	35.1
Toilet provision		
No toilet provision	54	70.1
Toilet less than 15m from shed	18	23.4
More than 15m from shed	5	6.5
Distance of house/community from shed		
Less than 15m	66	85.7
More than 15m	11	14.3
Distance of water tap from shed		
Less than 15m	43	55.8
More than 15m	34	44.2
Sleeping arrangement		
With blankets and mattress	48	62.3
Jute/sac/straw	22	28.6
Empty/Bare floor	7	9.1

### Qualitative findings: Focus group discussion with the adolescent girls

The results of the focus group discussion complemented the survey and provided in-depth insight into the lived experience of *Chhaupadi*. The thematic analysis suggested that most participants were restricted in terms of daily activities and nutritional intakes. Although *Chhaupadi* was practiced every month by the participants, they expressed willingness to discontinue it if given a choice.

#### Reasons for following *Chhaupadi*

All seven participants in the focus group discussion practiced *Chhaupadi* every month with the onset of menstrual period. Unanimously, the participants cited that family tradition was the main reason for following *Chhaupadi* and they believed that bad luck would shadow the family if the tradition were not followed. One participant stated:

“*I follow it because my family members and ancestors followed it and it will bring bad luck to my family if I don’t follow it and something bad will surely happen*.”

#### “Do’s and Don’ts” during menstruation

The participants were banished from the community and restricted from touching males, cattle, plants, food and water sources not allocated to them, as well as gods’ idols and sacred objects. They were also not allowed to go to shops or to the neighbors. For example, one participant stated, and others echoed similarly, that:

“*Immediately after I get my period*, *I leave the house for the shed*, *quietly without touching anything or anyone*. *My brothers will be sick if I touch them*. *The cattle will die*, *and the food will be rotten*. *There will be a death in the family if we don’t follow it*.*”*

The students shared that although they were permitted to attend school during their menstruation, they were not allowed to touch the water tap at school or eat food at the regular canteen and shops. They also went to and from school straight from the exiled living spaces used during menstruation while practicing *Chhaupadi*.

#### Nutritional restriction during *Chhaupadi*

All of the participants in the focus group discussion informed the interviewer that they were brought food by a female family member, usually a sister or mother, at the *Chhau* shed or other living space during menstruation. During *Chhaupadi*, girls were restricted from eating dairy products because they believed that it would cease the lactation of the cattle. One girl explained:

“*I am never given milk and milk products because if I drink milk while on my period*, *the cow will stop giving milk*.”

#### Willingness to follow *Chhaupadi*

One participant stated that she prefers to practice *Chhaupadi* since not practicing it would bring misfortune to her family. Except for her, all other participants said that they didn't like the practice of *Chhaupadi*, and that if given a choice, they would not follow it. Staying at the shed was boring for the girls and was accompanied by fear of physical and mental harm. One of the girls who was quite unhappy with the practice expressed:

“*I don’t like to stay at the shed. There is a constant fear of the animals, snakes, and scorpions. We hear about women being raped and abused while staying in the shed*”.

### Qualitative findings: Key informant interviews

The main themes that evolved from the key informant interviews were perceptions of *Chhaupadi*, the problems of *Chhaupadi*, the past and the present practice of *Chhaupadi*, and challenges on the way forward.

#### Perceptions of *Chhaupadi*

*Chhaupadi* was perceived as a long-standing tradition of banishing women and girls into the sheds or outside their homes during menses. It was deeply believed that women and girls are impure while menstruating; consequently, any objects touched by her at that time would be impure. Hence, the belief that women and girls should be isolated. The school teacher said:

“*People believed that menstruation is the period of impurity and anything touched by them while on period will be impure or waste the things too*. *Such belief has been in place from the ancient time or the time immemorial*”.

#### The problems

The key informants unanimously identified security and safety as the chief concern of the *Chhaupadi* practice. The female community health volunteer alleged:

“*The sheds are not secure and safe. They are small, with no proper ventilation, light, electricity and other necessities. There are many cases in which women have died of cold and suffocation. The things get worse in winter when the women light fires for heat inside the shed with no ventilation. Sanitation and hygiene is another problem*”.

#### The past and the present

According to the key informants, the practice of *Chhaupadi* has been changing slowly, and society has become softer in terms of enforcing the practice. Compared to past decades, changes have occurred such as decreased distance between the community and the shed, community movements to abolish the *Chhaupadi* practice, and permission to go to school and read. The representative from a local organization, who has been closely watching the practice for the past 13 years recalls:

*“People are slowly shifting the sheds near their homes*, *and some have also started to let the women live inside their homes while still living in isolation and not touching forbidden things*. *Slowly*, *people are being civilized*, *and they are decreasing the severity of the practice*. *Now*, *girls are allowed to go to schools even during their menstruation periods”*.

#### Challenges on the way

Strongly held traditional beliefs are the main challenges. Overcoming long-held traditions is difficult, especially if they are believed to be associated with misfortune. The representative from a local organization further adds:

“*The challenges we are facing in our organizations in the abolishment of Chhaupadi are that people have traditional values/beliefs which are difficult to change*. *People have the superstitious belief that letting menstruating women inside homes will bring misfortune to their family*. *The beliefs are so engrained that they believe that the recent disastrous earthquake in Eastern and Central parts (of Nepal) was due to the sin people have committed by letting menstruating girls and women in their homes”*.

#### Way forward

Key informants believed that increased education and a community movement to abolish the *Chhaupadi* tradition is needed to end the practice. Local community organizations and the local government are working in affected areas to abolish the practice. One local movement called “*Chhau goth bhatkau abhiyan*” led to destroying the *Chhau* sheds in this area. Additionally, emphasis has also been on empowering and educating women and girls as well as advocacy at different levels. Key informants hope that education, the empowerment of females, and the community movements will ultimately eradicate the practice from the community. The school teacher shared:

“*Adolescent girls understand the problems and consequences of the practice and are willing to bring the change*. *The practice of personal and menstrual hygiene even at Chhau shed had been increased so that they do not suffer from infections*. *Many organizations are working to abolish the practice and to educate women about menstrual hygiene*. *This is the hope and the future*.”

## Discussion

The current study found that all study participants practiced the taboo of untouchability during menstruation and the majority of the girls (n = 77, 72%) practiced exile, or *Chhaupadi*. The adolescent girls in our study faced many physical, psychological, and social problems and were restricted in terms of certain foods and other daily activities. The living spaces during *Chhaupadi* lacked basic neccessities. The internalized belief among the interviewed girls that not following *Chhaupadi* would bring misfortune to the family was the driving force of the practice.

The girls expressed sentiments that suggested they would rather not practice *Chhaupadi*, but Nepal’s patriarchial society sets distinct socialization patterns for girls: voicing of needs, concerns and opinions is discouraged and they are not given opportunities to make decisions [29]. Further, strong familial and community bonds means that rebellion is extremely rare and unlikely, so the girls follow the mandates imposed by their parents [29].

*Chhaupadi* is highly prevalent in far-western Nepal, especially in the districts of Achham [6]. *Chhaupadi* practice aligns with Hindu culture, which views menstruation as a “curse”, and menstruating woman as “impure”, thus menstruating woman are prohibited from usual religious ceremonies, including entering prayer rooms and the temples [30,31]. In Buddhist tradition, menstruation is seen as a natural bodily process; no general restrictions are placed on menstruating women, although some Buddhist temples forbid them to enter [3]. Similarly, most Christian sects today do not follow any specific rituals or regulations related to menstruation, although the Old Testament of the Bible indicates that a menstruating woman is impure [3]. Only a few, including Russian Orthodox Christians and Coptic Christians in Ethiopia continue to believe in menstrual taboos; women are restricted from church services during menstruation [3]. In Islam, a menstruating woman is considered ritually impure and is restricted from religious rituals such as sitting in a mosque, touching the Qur’an, daily prayers, and fasting during the month of Ramadan [3]. Both Islamic and Jewish religious law specifically forbid engagement in sexual intercourse during the days of menstruation [3]. While other religions have some taboos associated with menstruation, women are permitted to live in the home as usual and to eat and drink with the family [3]. However, notions of purity and pollution during menstruation are central to Hindu culture: all women during menstruation and childbirth are considered impure and thus restricted from participation in normal daily activities [3]. These restrictions are simply much harsher in regions practicing *Chhaupadi*, as women are banished to a shed even when the weather is freezing cold, restricted from eating and drinking with the family as usual, and excluded from key community activities.

We believe that religious beliefs may be further aggravated by deprivation and illiteracy. Of the total 75 districts in Nepal, Achham is the second least developed in terms of the Poverty and Deprivation Index, and the least developed in terms of the Socioeconomic and Infrastructural Development Index [32]. The overall literacy rate for ages 5 and above is 62%, with women’s literacy at only 47% [33]. We believe that poverty and deprivation coupled with illiteracy might explain the high prevalence of *Chhaupadi* practice in this district. Since disobeying *Chhaupadi* is believed to bring misfortune, people already struggling to meet their basic daily needs are more likely to abide by the superstition for the sake of avoiding any additional harm.

Our participants experienced several physical and psychological problems. We found diarrhea and dehydration, hypothermia, and reproductive and urinary tract infection to be common health problems among menstruating young women practicing *Chhaupadi*. Previous research has found that reproductive health problems such as burning micturition, abnormal discharge, itching in genital region, pain and foul-smelling menstruation were significantly higher among women of menstrual age who practiced *Chhaupadi* compared to those who did not [14]. Winter in the hilly areas of Nepal, such as Achham, is harsh; yet in our observation of the living spaces during *Chhaupadi*, we found that many girls lacked a mattress and warm blankets and rather slept on a rug or bare floor with sacks as cover. This may explain why hypothermia is a substantial problem among those practicing *Chhaupadi* [34, 35].

Our findings of social restrictions while practicing *Chhaupadi*, in terms of certain food and other daily activities, was also not surprising. In our study and other reports [6,36], participants were restricted from eating dairy products. Importantly, our focus group discussion revealed the local belief that cattle would cease lactating if menstruating women and girls consumed milk and milk products. Overall, such dietary restrictions may jeopardize the health of our participants and other adolescent girls in the area, as nutrition is key to healthy growth and development among adolescents [37]. According to Nepal’s annual household survey [33], on average, rural households in Nepal consumed milk or other dairy products 6.2 days a week, suggesting that milk is available. Additionally, limited studies conducted in Nepal show Nepalese adolescent girls with a high prevalence of malnutrition, with approximately 59% being undernourished [38,39]. While nutritional status is naturally dependent on the household’s ability to afford and access food, it is also likely that the poor nutritional status of Nepalese adolescent girls is further aggravated by *Chhaupadi*. In fact, in *Chhaupadi*-practicing areas, women have twice the prevalence of anemia and emaciation than in urban area [36].

The researchers were impressed to see that most of our participants were permitted to attend school and read books while menstruating. In the past, menstruating girls were expected to halt school attendance, since one Hindu belief is that ‘Sarswoti,’ the goddess of education, will become angry if a girl or woman reads, writes or touches books during her menstrual cycle. However, the number of girls attending school during menstruation has significantly increased recently [6], likely due to increased governmental and non-governmental focus on the education of girls to meet Millennium Development Goals [40]. We hope that this shift foreshadows increased relaxation of menstrual taboos concurrent with the increasing education of girls.

The sanitation and safety of the sheds is a major concern. In our study, we observed that an unhygienic livestock shed is a popular shelter during *Chhaupadi*. The shed did not have any provision for basic necessities such as ventilation/ windows, secure door locks, electricity, and toilet facilities. In general, many households in rural Nepal lack basic amenities [33]. However, in Achham district, approximately 48% of households had a toilet, 56% had access to a piped water supply, and 18% had electricity [20]. Therefore, the lack of basic facilities observed in the living spaces during *Chhaupadi* is not completely explained by poverty conditions in rural households; the girls were simply not allowed to access the amenities in their households during this time.

Safety was a major concern. Nine participants in our study were bitten by a snake during menstrual exile. The recurrent news of Nepali women and girls dying of snakebites and other causes while practicing *Chhaupadi* [41,42,43], although disheartening, seems unavoidable in the current context, given that the *Chhau* or livestock shed is isolated, exposed, dark and lacking a secure door lock. Moreover, medical treatment is inaccessible or delayed, and village shamans may be prefered for medical care [9,44]. Lastly, physical abuse, albeit not specifically rape, while in *Chhaupadi* was reported in the current study. Moreover, a 2011 UN report suggested that physical abuse generally, and rape specifically, are greatly underreported due to stigma [6].

The main strength of our study is its pioneering examination of the situation of Nepali adolescent girls practicing *Chhaupadi*. Our use of a mixed-methods approach allowed for triangulation of information from multiple sources, while a single, quantitative method may not have effectively explored the lived experiences of those practicing the taboo. This study was limited to adolescent girls and may not be representative of all women who practice *Chhaupadi*. Generalizability may be limited due to the use of purposive sampling, the inclusion of one specific village, and the inclusion of girls in school; however, we have no reason to believe these girls differ meaningfully from other girls in similar regions. Women’s literacy is low in Nepal (57%) [45], and lower in our study village (47%) [33]. All responses were self-reported by the participants; due to the feelings of insecurity, guilt, and humiliation associated with *Chhaupadi* [6], it is possible that some practices and/or conditions were underreported.

### Recommendations

As suggested by our key informant interviews and the UN’s (2011) report [6], women and girls’ education and awareness are seen as the key to abolish the tradition of *Chhaupadi*. *Chhaupadi* is a hindrance to gender equality in Nepal; as such, it should be central to the agenda among advocates of gender equality. Efforts to date have resulted in safeguarding gender equality and social inclusion through constitutional provisions; allocating reserved seats for women in the Constituent Assembly, civil service, and key decision-making positions; and launching programs to increase school enrollment among girls [40,46]. Nonetheless, Nepal is far from achieving gender equity. The government of Nepal, with support from international donors and programs such as the “100% Girls Scholarship Program” and the United Nations Girls’ Education Initiative, must continue its efforts to make schooling universal for all girls in Nepal, as set out in the Millennium Development Goals for 2000 and 2015 [40]. Public health professionals, with the Women’s Development Offices and their non-governmental organization (NGO) partners, should work with local schools to provide appropriate health education to both young girls and boys, emphasizing that the bodily functions of reproductive health are not shameful or dirty, but rather natural. Programs with extra instruction on safe menstrual hygiene practices have been conducted in some areas [11]; these should be systematically implemented throughout Nepal’s far-western region. Since *Chhaupadi* is a social problem with roots within community traditions, public health professionals should also continue to develop and implement educational interventions that include adult men and women at the community level [6]. While some progress has been made, as noted by the Key Informant, far more needs to be done.

Nepal has female community health volunteers whose work in advancing women’s health, particularly in rural areas, is praiseworthy [47]. Until menstruating women and girls are allowed to participate normally in the community, rather than being discouraged from seeking medical care during menstruation [9], regular visits to the sheds by female community health volunteers may be beneficial to monitor the health needs of the women and girls and provide basic health care during this time. Future research should target women practicing *Chhaupadi* during childbirth. Inclusion of school girls only may have impacted our findings as illiterate girls are more likely to follow the taboos and less likely to accept changes in traditions; thus, our results may be understated. Future research should consider the experience of adolescent girls who do not attend school.

It is evident that beliefs in *Chhaupadi* are so ingrained in the culture, especially in far-western Nepal, that abolishment does not seem plausible in the short term; the well-intentioned government regulation to ban the practice will take time to be implemented. Until then, public health professionals, whether governmental or non-governmental, should intervene with temporary measures to promote the health and safety of Nepalese women and girls practicing *Chhaupadi*. Simple interventions such as putting screens on windows and doorknobs/locks on doors could prevent the unfortunate incidents of physical abuse, rape and death due to animal bites. Likewise, provision of warm blankets, mattresses, food, water, sanitary pads and other basic necessities will help to ensure the basic needs of these young women and girls are met. As a temporary solution, we propose common community shelters to be built where all menstruating women and girls can reside together. These common facilities, well-ventilated and with locking doors and windows, would have all basic necessities including food preparation areas, piped water and toilets. They would not only prevent illness and death but also promote sanitation, health, and nutritional requirements. In a limited resource country like Nepal, financial constraints may be a challenge to meeting the requirements of those practicing *Chhaupadi*. Nevertheless, as long as *Chhaupadi* exists, there should be health and safety provisions made until its ultimate eradication.

In conclusion, the tradition of *Chhaupadi* is still common among adolescent school girls in far-western Nepal, even though it imposes physical and mental hardships and challenges fundamental human rights [6]. A brighter future for Nepali women and girls must be predicated upon the eradication of the practice of *Chhaupadi*.

## Supporting information

S1 Text Study tools in Nepali. (PDF)

S2 Text Unofficial English translation of study tools. (PDF)

S1 Dataset (XLSX)
