# Supplementary material for: Practice and lived experience of menstrual exiles (Chhaupadi) among adolescent girls in far-western Nepal
Source: PLoS One. 2018 Dec 10;13(12):e0208260. doi: 10.1371/journal.pone.0208260 (PMC6287853; doi:10.1371/journal.pone.0208260)
Supplement: S1 Text — (PDF) [file pone.0208260.s001.pdf]

S1 Text. Study tools in Nepali

सामाजिक-जनसंख्ययिक जानकारी:

१. नाम: ....
२. उमेर (वर्ष): ....
३. जात: ....
४. धर्म:
   क. हिन्दु   ख. बुधिस्ट   ग. ख्रिस्तियन   घ. मुस्लिम   ङ. अन्य
५. वैवाहिक स्थिति:
   क. विवाहित   ख. अविवाहित   ग. सम्बन्ध बिच्छेद   घ. अन्य
६. शैक्षिक योग्यता:
७. परिवारको मुख्य पेसा:
   क. कृषि   ख. वैदेशिक रोजगार   ग. व्यापार   घ. श्रम   ङ. जागिर
   च. अन्य
८. परिवारको मासिक कूल आय: .... /-
९. तपाइको पेसा:
   क. विध्यार्थी   ख. श्रमिक   ग. कृषि   घ. गृहिणी   ङ. जागिर
   च. अन्य

छाउपडी सम्बन्धि प्रश्न:

१. छाउपडी प्रथा अपनाउनु भएको छ?
   क. छ   ख. छैन
२. महिनावारी/नछुने हुदा कहाँ बस्ने गर्नुभएको छ?
   क. छुइकटेरो   ख. गोठ   ग. घर बाहिर/ खुल्ला स्थानमा   घ. घर भित्र छुट्टै कोठा वा स्थानमा   ङ. अन्य
३. महिनावारी हुदा कहाँ खाना खानुहुन्छ?
   क. बसेको स्थानमा   ख. घर भित्र   ग. घर बाहिरा
४. महिनावारि हुँदा साबिक जस्तो खाना खानुहुन्छ?
   क. खान्छु   ख. खादिंन

४.१. खानु हुदैन भने: कस्तो खाना खनुहुन्छ?

....

५. महिनावारि हुँदा के-कस्तो खाना खान दिईदैन?

क. माछा-मासु ख. दुध जन्य परिकार ग. साग-पात घ. फलफुल

६. महिनावारी हुँदा कति पटक नुहाउनु हुन्छ?

क. १ पटक २. २ पटक ३. ३ पटक ४. ३ पटक भन्दा बढि

७. महिनावारी हुँदा के प्रयोग गर्नुहुन्छ?

क. प्याड (sanitary napkin/pad) ख. घरेलु प्याड (pad) ग. कपडा घ. अन्य

८. प्याड (sanitary napkin/pad) अथवा कपडाको प्रयोग गर्नुहुन्छ भने कति समयमा फेर्नुहुन्छ?

क. ६ घण्टा ख. ६ घण्टा भन्दा कम ग. ६ घण्टा भन्दा बढि

९. Sanitary प्याड को व्यवस्थापन कसरि गर्नुहुन्छ?

क. पोको पारेर फ्याल्छु ख. जलाउछु ग. धोएर फेरी प्रयोग गर्छु घ. अन्य

१०. कपडा को प्रयोग गर्नुहुन्छ भने कसरि धुनुहुन्छ?

क. साधारण पानी ख. साबुन पानी ग. अन्य

११. कपडा कहाँ सुकाउनु हुन्छ?

क. घाममा ख. छाउ गोठ भित्र ग. घर भित्र घ. अन्य

१२. महिनावारी हुदा दिसा-पिसाब कहाँ गर्नुहुन्छ?

क. साबिक को सौचालय ख. छुट्टै सौचालय ग. खुल्ला ठाउँमा घ. अन्य

छाउपडी बस्दा **हुने समस्याहरु:**

१. छाउपडी बस्दा के-कस्ता सामाजिक समस्याहरु अथवा दुर्व्यवहार भोग्नु भएको छ?

क. बलात्कार ख. शारिरीक दुर्व्यवहार ग. चोरी घ. केहि भएको छैन

२. महिनावारि हुदा विध्द्यालय जानुहुन्छ?

क. जान्छु ख.जाँदिन

३. महिनावारी हुदा किताब पढ्नु हुन्छ?

क. पढ्छु ख. पढ्दिन

४. छाउपडी बस्दा तपाईलाई कस्तो महसुस हुन्छ?

क. कुनै पनि कुरा मा रुचि नलाग्नु　　ख. उदास हुनु

ग. रिस उठ्नु　　घ. निन्द्रा नलाग्नु

ङ. एक्लोपन महसुस हुनु　　च. केहि हुदैन

५. छाउपडी बस्दा चिसोको कारण कुनै समस्या परेको छ?

क. छ　　ख. छैन

५.१. छ भने, कुन समस्या छ/छन्? ______________________

६. छाउपडी बस्स्दा यी मध्य कुनै समस्या भएकाछन? (भएका सबै समस्या चयन गर्नुहोस्)

क. टाउको दुख्ने
ख. झाडा-पखालाको
ग. मुख सुक्खा हुनु
घ. प्यास लगिराख्ने
ङ. पिसाब गर्दा समस्या हुनु
च. कम पिसाब लाग्नु
छ. ढाड र तल्लो पेट दुख्नु
ज. पिसाब मा रगत देखिनु/ रातो पिसाब देखिनु
झ. ज्वोरो आउनु
ञ. केहि हुदैन
ट. अन्य

७. छाउपडी बस्दा छाला सम्बन्धि यी समस्याहरु भएकाछन? (भएका सबै समस्या चयन गर्नुहोस्)

क. बिविरा आउनु　　ख. चिलाउनु　　ग. छाला सुक्खा हुनु/ चर्किनु

घ. रातो फोका आउनु　ङ. दाग हरु देखापर्नु　　च. केहि हुदैन

८. छाउपडी बस्दा यी समस्या भएकाछन? (भएका सबै समस्या चयन गर्नुहोस्)

क. यौनि चिलाउनु　　ख. पिसाब गर्दा पोल्नु　ग. यौनि सुन्निने

घ. बाक्लो सेतो पानि बग्ने　ङ. केहि हुदैन

९. छाउपडी बस्दा यी समस्या भएकाछन ? (भएका सबै समस्या चयन गर्नुहोस्)

क. सर्प को टोकाई　　ख. किरा फट्यांग्रा को टोकाई

ग. जनावरको आक्रमण　　घ. अन्य

Observation checklist used for observation of living spaces during menstruation in Nepali:

१. छाउपडी बस्ने स्थानमा झ्यालको व्यवस्था:
   क. छ ख. छैन
२. झ्याल-ढोकामा चुकुल वा खापाको व्यवस्था:
   क. छ ख. छैन
३. छाउपडी बस्ने स्थानभित्र सर-सफाईको अवस्था:
   क. राम्रो ख. ठिकै ग. नराम्रो
४. छाउपडी बस्ने स्थानभित्र सुत्ने व्यवस्था:
   क. सिरत-डसना सहित ख. पराल/घाँस ग. जुट/बोरा

   घ. केहि पनि नभएको ङ. अन्य

५. छाउपडी बस्ने स्थानमा खानाको व्यवस्था:
   क. छ ख. छैन
६. छाउपडी बस्ने स्थानमा पिउने पानीको व्यवस्था:
   क. छ ख. छैन
७. छाउपडी बस्ने स्थानभित्र उज्यालो:
   क. छ ख. छैन
८. छाउपडी बस्ने स्थानभित्र बत्तीको व्यवस्था:
   क. छ ख. छैन
९. छाउपडी बस्ने स्थानमा शौचालयको व्यवस्था:
   क. छ ख. छैन
१०. शौचालय छ भने छाउपडी बस्ने स्थानबाट शौचालयको दुरी:
   क. १५ मि. भन्दा कम ख. १५ मि. भन्दा बढि
११. नजिकको घर/बस्ती बाट छाउपडी बस्ने स्थानको दुरी:
   क. १५ मि. भन्दा कम ख. १५ मि. भन्दा बढि
१२. छाउपडी बस्ने स्थानबाट नजिकको धारा/पानीको स्रोतको दुरी:
   क. १५ मि. भन्दा कम ख. १५ मि. भन्दा बढि
१३. छाउपडी बस्ने स्थानमा महिनावारी हुदा प्रयोग गर्ने कपडा सुकाउने ठाउँ:
   क. छ ख. छैन

Focus group discussion guides and probes in Nepali:

१. छाउपडी प्रथा अपनाउनु भएको छ?

२. छाउपडी प्रथाको बारेमा भन्नुहोस:

३. छाउपडी प्रथा किन अपनाउनु भएको हो?

४. छाउपडीको बेला के गर्नु हुन्छ र के गर्नु हुदैन ?

५. छाउपडीको बेला खान को बेवस्ता कसरि गर्नु हुन्छ?

६. छाउपडीको बेला के-के खानेकुराहरु बार्नु हुन्छ?

७. यो प्रथा बाट के-कस्ता समस्याहरु आउन सक्छन वा के-कस्ता समस्याहरु देख्नुभएको छ?

८. यो प्रथा तपाईलाई कस्तो लाग्छ?

    क) (राम्रो लाग्छ भने) किन राम्रो लाग्छ र भोलिका दिनमा पनि अभ्यास गर्नुहुन्छ?

    ख) (राम्रो लाग्दैन भने) किन राम्रो लाग्दैन र किन अभ्यास गर्दै हुनुहुन्छ?

Key Informant interview guides and probes in Nepali:

१. तपाईको नाम: ___________________________

२. तपाईंले काम गर्ने संस्थाको नाम? ___________________

३. तपाईले काम गर्ने संस्थामा तपाइँको पद के हो? _______________

४. यो ठाउँमा काम गर्नथाल्नु भएको कति वर्ष भयो?

५. यो क्षेत्रमा काम गर्नथाल्नु भएको कति वर्ष भयो?

६. छाउपडी परम्पराको बारेमा तपाईको के धारणा छ?

७. तपाईको ठाउँमा, पहिले र अहिले, छाउपडी प्रथाको अभ्यास कस्तो छ?

८. छाउपडी प्रथाको अभ्यास गर्दा किशोरीहरुमा के-कस्ता समस्या आएको देख्नु भएको छ?

९. छाउपडी प्रथाको विरुद्ध काम गर्दा के-कस्ता चुनौतीहरू आइपर्छन?

१०. तपाईंको विचारमा छाउपडी प्रथाको समस्या समस्या समाधान गर्न र अभ्यास समाप्त गर्न के गर्न सकिन्छ?
