# Supplementary material for: Practice and lived experience of menstrual exiles (Chhaupadi) among adolescent girls in far-western Nepal
Source: PLoS One. 2018 Dec 10;13(12):e0208260. doi: 10.1371/journal.pone.0208260 (PMC6287853; doi:10.1371/journal.pone.0208260)
Supplement: S2 Text — (PDF) [file pone.0208260.s002.pdf]

S2 Text. Unofficial English translation of study tools

Socio Demographic Information:

1. Name:

2. Age (years):

3. Ethnicity:

4. Religion:
   a) Hindu
   b) Buddhist
   c) Christian
   d) Muslim
   e) Others

5. Marital Status
   a) Married
   b) Unmarried
   c) Separated
   d) Others

6. Educational grade level:

7. Your family's primary occupation
   a) Agriculture
   b) Foreign employment
   c) Business
   d) Labor
   e) Jobs
   f) Others

8. Your family's monthly income:

9. Your occupation:
   a) Student
   b) Labor
   c) Agriculture
   d) Homemaker
   e) Jobs
   f) Others

Questions related to Chhaupadi

1. Do you practice Chhaupadi?
   a) Yes
   b) No

2. Where do you stay during menstruation?
   a) Chhau shed
   b) Cattle shed
   c) Courtyard
   d) Separate space inside home
   e) Others

3. Where do you eat during menstruation?
   a) Place where stayed during menstruation
   b) Inside home
   c) Outside home

4. Do you eat usual food during menstruation?
   a) Yes   b) No

   4.1. If you don't eat usual food during menstruation, explain what type of food do you eat?____

5. What foods are restricted during menstruation?
   a) Meat and poultry   c) Green vegetables
   b) Milk and dairy   d) Fruits

6. How many times during menstruation do you bath?
   a) Once   c) Three times
   b) Twice   d) More than three times

7. Material used to stem flow of menstrual blood
   a) Sanitary napkin/ pad   c) Clothes
   b) Homemade pad   d) Others

8. Frequency of changing pad or cloths
   a) Every 6 hours   c) More than 6 hours
   b) Less than 6 hours

9. How do you manage sanitary pads?
   a) Dispose   c) Reuse after washing
   b) Burn   d) Others

10. If you use clothes to stem flow of menstrual blood, how do you clean it?
    a) Water only   b) Soap and water   c) Others

11. Place for drying clothes during menstruation?
    a) Outside home in sunlight   c) Inside home
    b) Inside the living spaces   d) Others

12. Where do you excrete during menstruation?
    a) Regular toilet   c) Open Place
    b) Temporary toilet   d) Others

Problems experienced during *Chhaupadi*

1. What social problems or physical issues have you encountered during Chhaupadi?

a) Rape   c) Theft
b) Physical abuse   d) None

2. Do you attend school during menstruation?

a) Yes b) No

3. Do you read books during menstruation?

a) Yes b) No

4. How do you feel when you practice Chhaupadi?

a) Lack of interest
b) Depressed
c) Irritation
d) Sleep disturbance
e) Loneliness
f) None

5. Do you have problems due to cold during Chhaupadi?

a) Yes b) No

5.1. If you do have problems due to cold during Chhaupadi, what problems do you face? _______

6. Do you have any of these health problems during Chhaupadi? (select all that applies)

a) Headache
b) Diarrhea
c) Dehydration
d) Thirstiness
e) Problem with urination
f) Less frequent urination
g) Lower abdominal and back pain
h) Blood in urine
i) Fever
j) None
k) Others

7. Do you have any of these skin problems during Chhaupadi? (select all that applies)

a) Rashes
b) Itches
c) Dry skin/cracks
d) Red pustules
e) Scars
f) None

8. Do you have any of these problems during Chhaupadi? (select all that applies)

a) Vaginal inflammation
b) Burning during urination
c) Swollen vagina
d) Vaginal discharge
e) None

9. Do you have any of these problems during Chhaupadi? (select all that applies)

a) Snake bite
b) Insects bite
c) Animal attack
d) None

Observation checklist used for observation of living spaces during menstruation in English:

1. Availability of ventilation/windows in the living spaces:
   A. Yes B. No

2. Availability of lock in doors and windows:
   A. Yes B. No

3. Hygiene and sanitation inside the living spaces:
   A. Good B. Poor C. Average

4. Sleeping arrangements in the living spaces:
   A. With Blankets and Mattress B. Straw C. Jute/Sac/ D. Empty Floor E. Others

5. Availability of food in the living spaces:
   A. Yes B. No

6. Availability of drinking water in the living spaces:
   A. Yes B. No

7. Brightness inside the living spaces:
   A. Yes B. No

8. Availability of electricity in the living spaces:
   A. Yes B. No

9. Provision of toilet in the living spaces:
   A. Yes B. No

10. If toilet is available, distance of toilet from the living spaces:
    A. Less than 15m B. More than 15m

11. Distance of living spaces from nearest house/community:
    A. Less than 15m B. More than 15m

12. Distance of nearest water tap from the living spaces:
    A. Less than 15m B. More than 15m

13. Availability of place, in the living spaces, for drying clothes used to stem the flow of menstrual blood:
    A. Yes B. No

Focus group discussion guides and probes in English:

1. Do you follow Chhaupadi tradition? (if no then end the interview)
2. Tell me about the Chhaupadi tradition?
3. Why do you follow Chhaupadi?
4. During Chhaupadi what can you do or cannot do?
5. How do you manage food during Chhaupadi?
6. What food items you don’t eat while on Chhaupadi?
7. What are the problems of Chhaupadi you have faced or seen?
8. How do you feel about the Chhaupadi tradition?
    a. (Ask only to participants who like the Chhaupadi tradition) why do you like the Chhaupadi tradition, and will you continue to follow it in future?
    b. (Ask only to participants who don’t like the Chhaupadi tradition) If you don’t like the Chhaupadi tradition, why don’t you like it and why do you still follow it?

Key Informant interview guides and probes in English:

1. Your name? ________________________________
2. Name of the organization you work for? ___________
3. What is your designation in the organization?
4. How long have you been working in this local area?
5. How many years have you been working worked in this field?
6. What do you think of the Chhaupadi tradition?
7. What is the condition of Chhaupadi practice in your area in past and at present?
8. What problems have you seen due to the practice of Chhaupadi in adolescent girls?
9. What challenges do you face working against Chhaupadi?
10. In your opinion, what can be done to solve the problems of Chhaupadi and to eliminate the practice?
